# Effect of a liberal fluid fasting policy before surgery on the incidence of aspiration: a single centre retrospective observational study

**DOI:** 10.1016/j.bjao.2026.100578

**Published:** 2026-07-01

**Authors:** Marije Marsman, Maarten Derksen, Marloes Veltcamp Helbach, Judith A.R. van Waes, Teus H. Kappen, Evelien C. van der Hout, Maurice E. Pouw, Wilton A. van Klei

**Affiliations:** 1 Department of Anaesthesiology and Intensive Care Medicine, University Medical Centre Utrecht, The Netherlands; 2 Department of Anaesthesiology Meander Medical Centre, Amersfoort, The Netherlands; 3 Department of Anaesthesiology and Intensive Care Medicine, University Medical Centre Utrecht, Utrecht, The Netherlands; 4 Department of Information Technology, University Medical Centre Utrecht, Utrecht, The Netherlands; 5 Department of Pulmonology, University Medical Centre Utrecht, Utrecht, The Netherlands; 6 Department of Anesthesiology and Intensive Care Medicine, University Medical Centre Utrecht, Utrecht, The Netherlands; 7 Department of Anaesthesiology and Pain Medicine, Temerty Faculty of Medicine, University of Toronto, Toronto, ON, Canada

**Keywords:** aspiration, guidelines, implementation, liberal, preoperative fasting, regurgitation

## Abstract

**Background:**

In adults undergoing anaesthetic procedures, a preoperative fasting duration of 2 h for clear fluids is advised to minimise pulmonary aspiration risk. This fasting duration is under debate, because a shorter duration has been shown to improve patient wellbeing and seems not markedly to increase the aspiration risk. Although some hospitals already have adopted such a policy, large-scale studies addressing safety of this strategy are lacking. Therefore, the primary aim of this study was to assess the incidence of aspiration after implementation of a liberal fluid fasting policy.

**Methods:**

This observational single-centre cohort study was performed in a Dutch tertiary referral hospital after implementation of a liberal fluid fasting policy and included adults undergoing elective and urgent procedures under anaesthesia. The primary outcome was pulmonary aspiration, defined as documented visualisation of gastric content in the tracheobronchial tree below the vocal cords as seen by laryngoscopy, bronchoscopy, after tracheal suctioning with or without signs of respiratory distress or both. Secondary outcomes were regurgitation (visualisation of gastric content in the oral cavity or pharynx) or aspiration pneumonia. Risk factors for aspiration were identified using logistic regression analysis.

**Results:**

Between June 2019 and February 2025, 56 995 patients were evaluated with a recognised aspiration incidence of 3.7 (95% confidence interval [CI] 2.4–5.6) per 10 000 patients. The incidence of regurgitation was 25 (95% CI 21–29) per 10 000, and aspiration pneumonia occurred in 2.1 (95% CI 1.2–3.7) per 10 000. Median fasting duration for clear fluids was 63 min (inter-quartile range 24–116 min).

**Conclusions:**

A liberal fasting policy for clear fluids before elective and urgent surgery in adults had a higher associated incidence of aspiration compared with the previously reported incidence using a standard 2-h fasting policy. Because a liberal fasting policy has been shown to improve patient wellbeing, this study could contribute to the discussion among patients and clinicians to balance the benefits and risks of such a liberal policy.

**Editors’ key points**

• Allowing adult surgical patients to drink clear fluids until just before surgery may improve comfort, hydration, and reduce postoperative nausea and vomiting.
• Earlier studies using various fasting protocols, including the standard 2-h clear fluid cutoff, reported aspiration rates of 1.2 to 4.1 per 10 000.
• This large retrospective single-centre study evaluated aspiration risk with a liberal fasting policy allowing up to one glass of clear fluid per hour until arrival in the operating room.
• Protocol adherence was high: more than 75% of patients fasted less than 2 h for clear fluids. In a subgroup of elective general anaesthesia cases, median fasting time was an hour.
• Aspiration occurred in 21 of 56 995 patients, an incidence of 3.7 per 10 000 (95% confidence interval 2.4–5.6).
• This rate was higher than some previous reports using standard fasting guidelines and exceeded that considered acceptable by a patient advisory panel.
• The study provides useful real-world evidence on the risks of a liberal clear fluid fasting policy.

The goal of preoperative fasting is to minimise the risk of pulmonary aspiration in patients undergoing procedures under anaesthesia or procedural sedation. Current guidelines for adults recommend withholding solid food for 6 h and clear fluids for 2 h before anaesthesia induction in elective procedures and urgent procedures requiring surgery within 24 h.^1^, ^2^, ^3^ In adult patients with normal gastric function this duration results in minimal gastric volumes, with a reported incidence of aspiration between 1 and 4 per 10 000 procedures.^4^, ^5^, ^6^, ^7^ In children, it has been reported that a more liberal approach to fluid fasting is safe and improves patient wellbeing, resulting in a change in paediatric perioperative fasting guidelines.^8^, ^9^, ^10^, ^11^, ^12^, ^13^, ^14^, ^15^, ^16^, ^17^ In adults, there seems to be more reluctance to adopt a liberal fluid fasting policy. Although such a policy has been shown to improve patient wellbeing, large-scale studies to assess safety are lacking.^18^ Only a limited number of studies assessed the risks and benefits of a liberal fluid fasting policy, some of which are post-implementation reports of European clinical practices that have implemented a liberal fluid fasting policy despite the limited evidence in adults.^18^, ^19^, ^20^, ^21^, ^22^, ^23^, ^24^, ^25^, ^26^ A multicentre randomised study to assess safety would be ideal, but the excessively large number of patients required to detect relevant changes in aspiration incidence hinders the initiation of such a trial.

Without clear safety evidence, liberal fasting policies are unlikely to be widely adopted or reflected in guidelines. This impasse underscores the need for accurate estimates of safety measures from practices where a liberal fasting policy is standard of care.^27^ In a previous large observational cohort study, we found that the incidence of aspiration after implementation of a liberal fluid fasting policy was 2.4 (95% confidence interval [CI] 0.5–4.7) per 10 000, but given the rare occurrence of aspiration, this study was underpowered to prove safety.^18^ Therefore, the primary aim of the current study was to estimate the incidence of aspiration in a larger cohort of patients in whom a liberal fluid fasting policy was applied, allowing clear fluids until surgery. The secondary aims were to report on the incidence and identify risk factors for regurgitation and aspiration.

## Methods

### Patients

This retrospective observational cohort study included adult patients (≥18 yr) scheduled for elective or urgent procedures under general anaesthesia, locoregional anaesthesia (with and without sedation), procedural sedation only, and monitored awake procedures, at the University Medical Centre Utrecht (Utrecht, NL, USA), a tertiary referral hospital. Patients were included between June 2019 and February 2025, after local implementation of a liberal fasting protocol for clear fluids in the time period 2019–2021.^18^ Patients who had surgery between November 2023 and March 2024 were excluded, as the data were considered unreliable because of a hospital wide implementation of a new Electronic Medical Record (EMR) system. Patients requiring emergency intervention within 8 hr, those who were intubated before surgery, and obstetric patients were also excluded because the liberal fasting protocol was not applicable in these patient groups. Data from patients included between June 2019 and July 2021 were previously reported in a study comparing fasting duration and patient wellbeing between standard and liberal fluid policy.^18^ These data were reused in the current study to achieve a larger cohort of patients to estimate the incidence of aspiration under a liberal fluid policy.

The local medical ethics committee waived the need for informed consent, because of the retrospective nature of the study (University Medical Centre Utrecht Medical Research Ethics Committee 18–896/C,15-1-2019). The Strengthening the Reporting of Observational Studies in Epidemiology guidelines for reporting of observational studies were followed.

### Fasting policy in routine clinical care

According to the liberal fasting policy, drinking clear fluids was allowed and encouraged until arrival in the operating room, up to a maximum of one glass (150 ml) per hour. Nurses were instructed to offer pre-emptive analgesics with one glass of water (150 ml) 30–60 min before the anticipated procedure start. Solid food was not allowed for 6 h before surgery.

### Data collection

All data were documented during daily care and obtained from the EMR systems (Chipsoft®, The Netherlands and Anstat®, Carepoint, The Netherlands) as described previously.^18^ Free text records within both EMRs were systematically searched for the keywords aspiration, regurgitation, vomiting, bile and food, and related words. In addition, the hospital’s critical incident reporting system was searched for the keywords aspiration and regurgitation. Subsequently, three authors (MM, MD, MV) assessed and double-checked the medical records of the patients with suspected regurgitation or aspiration, to confirm the likelihood of true occurrence of regurgitation or aspiration based on the medical record notes. If the occurrence of regurgitation was unclear, this was discussed until agreement. To adjudicate the diagnosis of pneumonia, the medical records of patients with suspected aspiration pneumonia were also assessed by an independent pulmonologist (EH).

### Outcomes

The primary outcome was aspiration, defined as documented visualisation of gastric content in the tracheobronchial tree below the vocal cords as seen by laryngoscopy, bronchoscopy, after tracheal suctioning with or without signs of respiratory distress or both. In addition, patients in whom signs of perioperative distress were documented, such as reduced oxygen saturation, increased ventilatory frequency, coughing, or wheezing after a registered regurgitation, were classified as having aspirated. Finally, patients developing aspiration pneumonia after regurgitation (with or without subsequent tracheal suctioning or inspection) were also classified as having aspirated. A classification ‘unverified aspiration’ was used for patients with observed regurgitation, but in whom laryngoscopy, bronchoscopy or tracheal suctioning was not performed, and who had no signs of perioperative respiratory distress or postoperative pneumonia, as a result of which (mild) aspiration could not be completely ruled out.

Secondary outcomes were regurgitation, aspiration pneumonia and fasting duration for clear fluids and risk factors for regurgitation and aspiration. Regurgitation was classified as visualisation of gastric content in the oral cavity or pharynx, regardless of aspiration occurrence. Aspiration pneumonia was defined as pneumonia within 24 h after surgery, diagnosed by the attending physician based on clinical symptoms of respiratory distress and fever, and if available by a postoperative chest radiograph demonstrating a new infiltrate that did not exist before surgery and that developed within 24 h after surgery. This diagnosis was subsequently retrospectively reviewed and adjudicated by a pulmonologist. The duration of fasting for clear fluids was defined as the time between the last documented intake of more than half a glass of fluids and induction of anaesthesia. In addition, risk factors for regurgitation and aspiration were assessed.

### Sample size

Previous studies including patients who fasted according to current guidelines, reported an incidence of aspiration between 1.2 and 4.1 per 10 000.^4^^,^^5^^,^^18^ In our previous study we found an incidence of aspiration of 2.4 (95% CI 0.5–4.7) per 10 000 in patients with a liberal fasting policy, as compared with 1.7 (95% CI 0.6–2.7) per 10 000 in patients with a standard fasting policy.^18^ As this estimate was still quite uncertain despite the reasonably large sample size in the previous study, in the current study we aimed to provide a more precise estimate of the aspiration risk in patients following a liberal fluid fasting policy. A patient advisory panel, consisting of four patients with experience in anaesthesia and surgery, was asked for input regarding what aspiration risk they considered relevant, weighing the risks and benefits of a liberal fasting policy. They considered that roughly a two-fold increase in the incidence of aspiration would be acceptable from a patient’s perspective if shorter liquid fasting would otherwise increase wellbeing. Specifically, patients considered a lower incidence of postoperative nausea and vomiting most important. Based on our previous study, we assumed the aspiration incidence in patients following a liberal policy would be around 2.0 per 10 000. With this assumed incidence, a sample size of 55 000 would result in an upper boundary of the 95% CI of 3.5 per 10 000, that is, approximately two times the incidence of 1.7 per 10 000 we previously found in the population fasting according to the current guidelines.

### Analysis

Baseline characteristics were presented as means, medians or percentages where appropriate. Normality of continuous variables was assessed through Q-Q plots and histograms, depending on their distribution. The incidences of aspiration, regurgitation and aspiration pneumonia were presented as numbers (95% CI) per 10 000 patients. The secondary outcome fasting duration was not normally distributed and therefore presented as a median.

Risk factors for regurgitation and aspiration were analysed using univariable logistic regression to obtain odds ratios (ORs) with 95% CI. A subgroup analysis was performed including only patients scheduled for elective procedures under general anaesthesia, based on scientific interest in aspiration risk specifically in these patients.^28^, ^29^, ^30^

Statistical analyses were performed with IBM SPSS Statistics for Windows (Version 29.0.1, IBM Corp., Armonk, NY, USA).

## Results

In total, 56 995 patients were included. The mean age was 56 yr (standard deviation 18) and 22 991 patients (49%) were women (Table 1). Median fasting duration in the whole cohort was 63 min (inter-quartile range [IQR] 24–116 min). There were some missing data, mainly for smoking (29%), a history of diabetes (16%) and fasting duration for all procedures (25%) (Table 1).

**Table 1:** Baseline characteristics. Figures are numbers (%) of patients, unless stated otherwise. GLP-1, glucagon-like peptide-1; sd, standard deviation. ∗Obesity was defined as a BMI ≥30 kg m^−2^. ^†^Smoking was defined as currently smoking. ^‡^Alcohol use was defined as drinking any alcohol daily. ^¶^Diabetes included patients with oral antidiabetics, insulin or both. ^§^ASA classification is physical status according to the ASA. ^||^Urgent surgery was defined as needing surgery between 8 and 24 h.

	Cohort (*n*=56 995)
Mean age in yr (sd) (min 18 max 101)	57 (18)
Female sex	27 777 (48.7)
Male sex	29 218 (51.3)
Length (cm, sd)	173 (12)
Missing	1207 (2.1)
Weight (kg) (sd)	79 (18)
Missing	1078 (1.9)
Mean BMI in kg m^−2^ (sd)	26.1 (5.1)
Missing	1592 (2.8)
Obesity∗	10 530 (18.5)
Missing	1592 (2.8)
Smoking^†^	7482 (13.1)
Missing	16,864 (29.4)
Alcohol use^‡^	26 220 (46.0)
Missing	4954 (8.7)
History of diabetes^¶^	5946 (10.4)
Missing	916 (16.2)
Use of GLP-1 agonist	215 (0.4)
ASA classification^§^	
1	5099 (8.9)
2	21 512 (37.7)
≥3	22 400 (39.3)
Missing	7984 (14.0)
Urgent surgery^||^	3726 (7.2)
Anaesthesia technique	
General anaesthesia	40 256 (70.7)
Procedural sedation	14 128 (24.8)
Peripheral nerve block	1254 (1.4)
Spinal anaesthesia	1664 (2.2)
Monitored awake procedure	357 (0.6)
Missing	208 (0.4)
Airway management in general anaesthesia	
Tracheal tube	31 528 (78.3)
Supraglottic airway/mask ventilation	7577 (18.8)
Unknown	1152 (2.9)
Surgical specialty	
Interventional cardiology	3445 (6.0)
Cardiothoracic surgery	3611 (6.3)
Endoscopy	7523 (13.2)
Head and neck surgery	6971 (12.2)
Gynaeco-urology	6842 (12.0)
General surgery	6040 (10.6)
Neurosurgery	4196 (7.4)
Ophthalmology	4861 (8.5)
Orthopaedics	3116 (5.5)
Psychiatry and interventional pain medicine	2873 (5.1)
Pulmonology interventional	1009 (1.8)
Reconstructive surgery	1912 (3.4)
Radiology interventional	2005 (3.5)
Vascular surgery	2570 (4.5)

The primary outcome aspiration occurred in 21 patients, resulting in an overall incidence of 3.7 (95% CI 2.4–5.6) per 10 000 (Table 2).

**Table 2:** Primary and secondary outcomes. Figures are numbers (%) of patients, unless stated otherwise. EHR, electronic health record; EMR, electronic medical record; IQR, inter-quartile range. ∗Patients with observed regurgitation, but in whom laryngoscopy, bronchoscopy or tracheal suctioning was not performed, and who had no signs of perioperative respiratory distress or postoperative pneumonia, as a result of which (mild) aspiration could not be completely ruled out. †The pulmonologist was uncertain about the diagnosis aspiration pneumonia based on the EHR recordings. ^‡^Calculated for patients where fasting duration was recorded in the EMR. Number of missing for fasting duration was 14 229 (25%).

	Cohort (*n*=56 995)	95% Confidence interval
Incidence of aspiration		
Yes	21 (0.037)	0.023–0.056
Unverified∗	100 (0.18)	
Incidence of regurgitation	140 (0.25)	0.21–0.29
Incidence of aspiration pneumonia		
Yes	10 (0.018)	0.001–0.003
Uncertain^†^	2 (0.004)	0.001–0.013
Median fasting duration (min) (IQR)^‡^	63 (24–116)	

With regard to the secondary outcomes, regurgitation was observed in 140 patients, resulting in an incidence of 25 (95% CI 21–29) per 10 000. Regurgitation occurred during induction, maintenance and emergence in 40 patients (29%), 80 patients (57%) and 20 patients (14%), respectively. Nineteen of the 140 patients with regurgitation (14%) did not aspirate and in 100 (71%) it was unverified if they aspirated, because inspection or suctioning of the trachea was not performed or not documented, as a result of which aspiration could not be completely ruled out. However, as none of these 100 patients experienced respiratory complications during or after anaesthesia, any possible aspiration would have been minimal. The secondary outcome aspiration pneumonia was diagnosed with certainty in 10 of the 21 patients (48%) who aspirated, and in two patients this diagnosis was uncertain. For the analysis, these two patients were considered to have had a pneumonia, resulting in an incidence of aspiration pneumonia of 2.1 (95% CI 1.2–3.7) per 10 000 patients. Table 3 describes the 12 patients with aspiration pneumonia. The aspirate of six patients contained not only liquids, but also solids. Four patients were multi-trauma patients requiring surgery in the course of several days after the trauma. Another patient had developed ileus secondary to systemic infection. Two patients aspirated probably because of an obstructive tumour in their upper gastrointestinal tract. The aspiration pneumonia resulted in delayed hospital discharge in six patients, unexpected ICU admission in five patients and may have contributed to death in one patient.

**Table 3:** Description of patients with an aspiration pneumonia. The diagnosis of aspiration pneumonia was unsure in patients 3 and 11. Unexpected ICU stay in patient 1, 2, 5, 8 and 9. Patients 1, 2, 3, 5, 6 and 9 had prolonged hospital stay because of aspiration pneumonia. An aspiration pneumonia may have contributed to death in patient 1. ESD, endoscopic submucosal dissection; NG, nasogastric; O_2_, oxygen; PEG, percutaneous endoscopic gastrostomy; TPV, total parenteral nutrition. ∗ASA classification is physical status according to ASA.

No	Yr	Age (yrs)	Sex	ASA∗	Anaesthesia technique	Phase of anaesthesia	Procedure	Fasting duration (min)	Amount of last fluid intake	Risk factors for regurgitation	Description event
1	2020	68	Male	3	General anaesthesia	Induction	Wound debridement	23	½ glass	Alcohol abuse. Urgent surgery because of infection of spondylodesis with systemic infection Preoperative (unrecognised) ileus	Aspiration during induction. After surgery the diagnosis ileus was made. Postoperative extubation, but subsequent reintubation because of respiratory insufficiency and admission to ICU. Infiltrate on chest X-ray. Antibiotics started. 2 days later emergency surgery because of progressive ileus, leading to septic shock. Patient died after 14 days because of multiple organ failure.
2	2020	61	Male	2	General anaesthesia	Induction	Fixation of acetabulum	50	1 glass	Daily alcohol use. Urgent multi trauma patient. Preoperative opioid use. Preoperative (unrecognised) gastroparesis	Aspiration of liquids and solids during induction. Postoperative ICU admission. Bronchoscopy for removal of remaining solids from the lungs. Infiltrate on chest X-ray. Antibiotics started. Discharge from ICU 2 days later and full recovery.
3	2020	30	Male	?	General anaesthesia	Induction	Pelvic fixation	129	½ glass	2 days after pelvic trauma and pulmonary contusion. Preoperative opioid use and nausea. Obesity	During induction regurgitation. Suctioning of oropharynx with subsequent endotracheal intubation. Unclear if tracheal suctioning was performed. Postoperative hypoxia. Unclear, based on symptoms and CT, if this was caused by worsening of the pulmonary contusion in combination with pain and anaesthesia or because of aspiration pneumonia. Antibiotics already preoperatively started because of complicated fractures.
4	2022	56	Male	3	Procedural sedation	Maintenance	Pacemaker implantation	unknown	unknown	None	Vomiting during procedure under procedural sedation. Suctioning of vomit. Aspiration unclear, but postoperative need for 2 L of oxygen. chest X-ray showed consolidation in right lower lobe. Antibiotics started. Discharge to home after 1 day with oral antibiotics.
5	2021	58	Male	3	General anaesthesia	Induction	Fixation femur and tibia	72	½ glass	4 days after multi trauma with multiple fractures and spleen laceration. Preoperative opioid use.	Patient regarded as fasted. During induction aspiration of black fluids with solids. After endotracheal intubation difficult ventilation with high pressures and high oxygen need. Subsequent bronchoscopy with suctioning of aspirate. Decision to cancel procedure and transfer to ICU. Infiltrate on CT-scan. Antibiotics started. ICU stay for 10 days. In hindsight preoperative gastroparesis and ileus.
6	2021	55	Female	3	General anaesthesia	Induction	Suprapubic catheter placement	55	1 glass	Preoperative opioid use: intrathecal morphine.	Last intake solids >21 h ago. After induction placement of supraglottic airway. Subsequent vomiting of fluids and ‘cheesy’ solids. Tracheal suctioning of yellow substances. Perioperative high oxygen need. Postoperative need for 5 L of oxygen. chest X-ray showed consolidation in left and right lower lobe. Antibiotics started.
7	2024	60	Female	3	Procedural sedation	Maintenance	PEG placement by gastroscopy	Nil per mouth policy already for 3 days	None	Gastroparesis because of metastasised cancer, for which already TPV was started, Nausea preoperative	Before procedure suctioning of stomach through NG tube. After introduction gastroscope again suctioning of stomach and solids and fluids in stomach. During procedure regurgitation, possibly duodenum fluids. At recovery increased O2 need. Antibiotics started. On chest X-ray consolidates right lung. Couple of days complaints of dyspnoea. No longer hospital stay than expected for procedure.
8	2024	32	Female	1	General anaesthesia	Maintenance	Arthrodesis wrist	90	Unknown	none	Coughing during closure of wound with subsequent regurgitation of gastric juices with subsequent severe bronchospasm and switch from supraglottic airway to endotracheal intubation. 1 night on ICU. Infiltrate on chest X-ray. No antibiotics started. After 2 days hospital discharge with some complaints (as planned).
9	2025	79	Male	3	General anaesthesia	Induction	Jejunum fistula for enteral feeding	61	Unknown	Obstructive oesophageal cancer, diabetes with oral medication	During induction regurgitation with aspiration. After endotracheal intubation suctioning of trachea and bronchoscopy to remove as much aspirate as possible. 1 Night ICU and antibiotics started. and prolonged stay in hospital.
10	2024	51	Male	2	Procedural sedation	Maintenance	ESD	unknown	Unknown	Difficult procedure gastroenterologist	Regurgitation of gastric juices with aspiration during procedure with low saturation for which conversion to general anaesthesia with endotracheal intubation. Antibiotics started for 5 days. After 1 day no complaints of dyspnoea, only some coughing. On chest X-ray infiltrate. No prolonged hospital stay.
11	2024	40	Male	2	General anaesthesia	Induction	Fixation of pelvic fracture	145	Unknown	Patient, 2 days after multitrauma with rib fractures, pneumocele and pleural effusions. Opioid use	Preoperative already respiratory compromised because of polytrauma. Vomiting after induction, suctioning of oropharynx and subsequent endotracheal intubation. Bronchoscopy with suctioning of all ostia. Intraoperative hypoxemia. Antibiotics started. After 1 day saturation as preoperative, some coughing. No longer hospital stay. Uncertain diagnosis of pneumonia.
12	2024	66	Male	3	Procedural sedation	Induction	Bronchoscopy	413	Unknown	Chronic opioid use	During bronchoscopy gastric fluids below vocal cords, which were suctioned away. Many abnormalities during bronchoscopy, as expected based on indication. Intraoperative no desaturations. Postoperative start of antibiotics. Discharge home same day.

Univariable regression analyses showed that a history of diabetes was a risk factor for regurgitation (OR 1.7, 95% CI 1.1–2.6), and endoscopic procedures (OR 2.5, 95% CI 1.7–3.6) (Table 4). Regarding aspiration, only urgent procedures were associated with aspiration in univariable regression analysis (OR 3.9, 95% CI 1.1–14).

**Table 4:** Univariable regression analysis for risk factors for regurgitation. CI, confidence interval; OR, odds ratio. ∗Obesity was defined as a BMI ≥ 30 kg m^−2^. ^†^Diabetes included patients with oral antidiabetics, insulin or both. ^‡^ASA classification is physical status according to ASA. ^¶^Urgent surgery was defined as needing surgery between 8 and 24 h.

	Unadjusted OR	95% CI
Age (per yr)	0.99	0.99–1.00
Female sex	0.84	0.69–1.35
Obesity∗	1.39	0.95–2.06
History of diabetes^†^	1.66	1.06–2.59
ASA classification^‡^		
1	reference	
2	1.5	0.77–2.92
≥3	1.1	0.58–2.25
Urgent surgery^¶^	1.17	0.61–2.2
Endoscopy	2.45	1.69–3.57
General anaesthesia	0.62	0.44–0.87

In a subgroup of 34 656 patients who underwent elective procedures under general anaesthesia, median fasting duration was 61 min (IQR 26–103 min). Aspiration occurred in 12 patients, resulting in an incidence of 3.5 (95% CI 2.0–6.1) per 10 000. Regurgitation was observed in 73 patients (incidence 21 [95% CI 17–26] per 10 000), and aspiration pneumonia in seven patients (incidence 2.0 [95% CI 1.0–4.2] per 10 000).

## Discussion

Application of a liberal fasting protocol for clear fluids in adult surgical patients resulted in an incidence of pulmonary aspiration of 3.7 (95% CI 2.4–5.6) per 10 000 patients. Adherence to the liberal fasting protocol was good, with more than 75% of patients having a fasting time for clear fluids under 2 h. The incidence was slightly above the incidence considered to be acceptable by a patient advisory panel (a two-fold increase, i.e. 3.4 per 10 000). Whether or not this observed increase in a rare but potentially severe outcome is acceptable to patients and clinicians, given the potential benefits of better hydration and mental state in many patients, is a matter of debate. Nonetheless, the increased incidence of both aspiration and aspiration pneumonia were close to what might be considered clinically relevant.

As the CIs of the incidence estimates are wide, and given this very rare outcome, we realise that, even with the inclusion of 56 995 patients, this study was still underpowered to obtain a precise estimate of the actual risk. However, when we take the results at face value, there are several things to consider. Among the 12 patients with aspiration pneumonia, nine had risk factors for delayed gastric emptying and it is unclear to what extent the liberal fluid policy contributed to aspiration in these patients, as four of them had a fasting duration of more than 2 h. Aspiration possibly could have been prevented with precautions, such as increasing fasting duration, performing gastric ultrasound, applying rapid sequence induction or both, although guidelines and literature are inconclusive which policy is best in these patients.^1^^,^^3^^,^^31^, ^32^, ^33^ These cases also reveal how difficult it can be for anaesthesiologist to recognise gastroparesis and adjust fasting duration for patients. It is also a subject of debate which fasting duration may be appropriate for patients with risk factors for delayed gastric emptying, such as glucagon-like peptide-1 use.^1^^,^^3^^,^^31^^,^^34^, ^35^, ^36^, ^37^ For example, the ASA states that a 2-h fasting policy for clear fluids can only be recommended to healthy patients, where the European Society of Anaesthesiology and Intensive Care guideline seems more liberal with regard to comorbidities.^1^^,^^3^

Then what to recommend? Comparable to the fasting guidelines for procedural sedation, a policy could be considered based on risk factors for aspiration, such as urgency, procedure characteristics, and risk factors for delayed gastric emptying.^38^ A standard 2-h fasting policy for clear fluids may be implemented in hospitals performing many procedures that are associated with a higher risk for aspiration and where patients with risk factors for aspiration are anaesthetised or both, whereas hospitals performing mainly elective low-risk procedures may implement a liberal fluid fasting policy as implemented in our hospital.

The reported benefits of a liberal fasting policy for patients are increased satisfaction second to a lower postoperative nausea and vomiting risk, less feelings of thirst, reduced anxiety and reduced headache.^18^, ^19^, ^20^^,^^23^^,^^39^, ^40^, ^41^, ^42^, ^43^, ^44^ For example, two recent studies in women scheduled for caesarean section showed that a liberal fluid fasting policy decreased fasting duration from 8 h to 1 h, improved comfort, and reduced thirst, nausea and anxiety.^19^^,^^44^ A quality-improvement study reported that implementation of a liberal fluid policy reduced fasting duration to 1.4 h and improved patient satisfaction.^43^ Another study, comparing liberal fluid fasting with standard fluid fasting showed reduced thirst and a decreased incidence of headache.^25^ Aspiration did not occur in any of these four studies, but they were all small-sized and therefore underpowered to assess these risks.

The strength of the present study is the quantity of data providing evidence on the safety of a liberal fluid policy. The results can stimulate debate among patients and policy makers to balance the benefits and risks of a liberal fluid policy, and may serve as a basis for a multicentre study in adults, comparable to the NIKS and EUROFAST study in paediatric anaesthesia, which is needed to provide more definite answers to remaining questions about safety and risk factors for aspiration. Our study has some important limitations that should be addressed. First, although this is the largest study in adults that has been performed on this topic, as the incidence of aspiration was somewhat higher than expected, it still appeared underpowered to prove comparable safety to the more classical 2-h fasting time. Next, over- and underreporting of regurgitation and aspiration could have been possible because of the retrospective study design. However, implementing a liberal fluid policy ahead of guidelines made anaesthesiologists vigilant about regurgitation, decreasing the chance for underreporting. Further, as this study was performed in a tertiary referral hospital with complex cases, resulting in a relatively high percentage of patients with risk factors for delayed emptying or urgent procedures, the results may not be generalisable to other hospitals caring for less complex patients. This limits the generalisability of our study, but at the same time underscores the need for a large multinational study. There were 25% missing data with regard to fasting duration, because of the voluntary nature of the nurses to register last intake. Although this percentage could be considered as high, other studies analysing large numbers of patients, such as the EUROFAST and NIKS study, only provided fasting duration for cases with aspiration.^13^^,^^23^^,^^45^

In conclusion, a liberal fasting policy for clear fluids before elective and urgent surgery had a higher incidence of aspiration as compared with the previously reported incidence using a standard 2-h fasting policy.^18^ Because a liberal fasting policy has been shown to improve patient wellbeing, this study contributes to the discussion among patients and clinicians to balance the benefits and risks of such a policy before elective surgery.

## Authors’ contributions

Conception and design: MM, JW, TK,WK

Data acquisition: all autors

Data analysis and interpretation: all authors

Drafting the manuscript: all authors

## Declaration of Generative AI and AI-assisted technologies in the writing process

No AI-assisted technologies were used in the writing process.

## Funding

Departmental resources.

## Declaration of interests

The authors declare that they have no conflicts of interest.
